# Effects of Spatial Subsidies and Habitat Structure on the Foraging Ecology and Size of Geckos

**DOI:** 10.1371/journal.pone.0041364

**Published:** 2012-08-10

**Authors:** Amy A. Briggs, Hillary S. Young, Douglas J. McCauley, Stacie A. Hathaway, Rodolfo Dirzo, Robert N. Fisher

**Affiliations:** 1 Department of Biology, Stanford University, Stanford, California, United States of America; 2 Western Ecological Research Center, U.S. Geological Survey, San Diego Field Station, San Diego, California, United States of America; University of Arizona, United States of America

## Abstract

While it is well established that ecosystem subsidies—the addition of energy, nutrients, or materials across ecosystem boundaries—can affect consumer abundance, there is less information available on how subsidy levels may affect consumer diet, body condition, trophic position, and resource partitioning among consumer species. There is also little information on whether changes in vegetation structure commonly associated with spatial variation in subsidies may play an important role in driving consumer responses to subsidies. To address these knowledge gaps, we studied changes in abundance, diet, trophic position, size, and body condition of two congeneric gecko species (*Lepidodactylus* spp.) that coexist in palm dominated and native (hereafter dicot dominated) forests across the Central Pacific. These forests differ strongly both in the amount of marine subsidies that they receive from seabird guano and carcasses, and in the physical structure of the habitat. Contrary to other studies, we found that subsidy level had no impact on the abundance of either gecko species; it also did not have any apparent effects on resource partitioning between species. However, it did affect body size, dietary composition, and trophic position of both species. Geckos in subsidized, dicot forests were larger, had higher body condition and more diverse diets, and occupied a much higher trophic position than geckos found in palm dominated, low subsidy level forests. Both direct variation in subsidy levels and associated changes in habitat structure appear to play a role in driving these responses. These results suggest that variation in subsidy levels may drive important behavioral responses in predators, even when their numerical response is limited. Strong changes in trophic position of consumers also suggest that subsidies may drive increasingly complex food webs, with longer overall food chain length.

## Introduction

Spatial subsidies, or the movement of nutrients or energy between ecosystems, can have substantial impacts on the abundance and community composition of primary producers and consumers, ultimately leading to large scale alterations in food webs and changes in ecological processes [1]–[3]. These subsidies have been shown to affect consumer abundance across a variety of consumer and ecosystem types [4]–[7], ranging from large vertebrate predators in desert systems [8] to microbial communities in temperate salt marsh systems [9]. The strength of response to variation in subsidy level in terms of consumer abundance has been shown to decline with trophic level of the consumer, with secondary consumers having less marked numerical response to subsidies than herbivores and detritivores [10]. This reduction in response for higher consumers is not surprising, considering their tendency to have longer generation times, lower fecundity, and more flexible diets [1]. Additionally, food and nutrient resources are often not the primary limiting factors of predator population sizes. Social limitations like territoriality and mate availability can regulate population densities at levels below the resource-determined carrying capacity [11], [12]. Compensation from other consumers may also contribute to lower numerical responses of predators to allochthonous inputs (i.e. [3]).

The aforementioned evidence has led to the idea that the importance of subsidies to consumers attenuates at higher levels of the food web [10], [13], [14]. However, a lack of observed numerical response by predators does not necessarily indicate that subsidy additions are not important to predator populations. There are multiple other avenues by which predators may respond to subsidy inputs. They may, for example, exhibit changes in behavior (i.e. shifts to subsidized foraging areas or subsidized diet items), instead of, or in addition to, changes in abundance, particularly in regions where subsidies are episodic [15]. Likewise, while slow reproduction or territoriality may prohibit numerical responses, subsidized consumers may show increased body size, fecundity, or other fitness metric [7], [16]. There may also be shifts of resource partitioning and dominance among different predator species that coexist in the same habitat but have differential reliance on subsidized food sources. Small changes in any of these factors may have large impacts on prey population, food web structure (i.e. food chain length, or connectivity among nodes in the food web), and ecosystem processes [17]–[19]. Moreover, subtle changes in consumer behavior, diversity, or abundance as a response to subsidy inputs may accumulate across a food web; such changes may be detectable only or primarily in higher consumers [20]. Since increased nutrient levels can increase food chain length and complexity [21], [22], we might expect to see higher trophic position for predators in more productive, subsidized food webs [23].

In addition to the effects of spatial subsidies on consumers, shifts in structure and complexity of habitat, whether caused by natural gradients, or anthropogenic disturbance can also cause changes in abundance and behavior of consumers, and alter patterns of competition and resource partitioning [24]–[26]. Changes in habitat structure (i.e. stem density, habitat complexity) and plant community composition have been shown to have significant effects on consumer abundance, body condition, competition and resource partitioning in several predators, including among various lizard species [24], [26], [27]. Such structural changes can also broadly alter food web topology, i.e. the distribution of connections between species in the food web with each other [28]. While changes in plant community composition and structure are very common and general across differentially subsidized systems [29]–[31], the importance of subsidy-related changes in habitat complexity to consumers has not been explicitly considered, as research has tended to focus on direct bottom-up effects of subsidies on consumers [32].

This work seeks a better understanding of both the effects of spatial variation in subsidy levels on predators, and the mechanisms by which these effects are generated. The first goal of the study was thus to identify the types and magnitudes of consumer responses across a subsidy gradient. To do this, we examined changes in abundance, diet, trophic position, and body condition of two gecko species - the parthenogenetic *Lepidodactylus lugubris*, and a recently characterized sexual species of *Lepidodactylus*, hereafter referred to as *L.* sp. nov. [33], [34] - across a strong gradient of seabird subsidies on islets of Palmyra Atoll, Central Pacific. These gecko species often co-occur and have similar morphological and ecological characteristics, although *L.* sp. nov. appears to be more associated with coastal habitats.

The second goal of the study was to understand if structural changes associated with varying subsidy levels can also explain observed responses to this subsidy gradient. In this system, decreases in subsidy input are strongly associated with changes in plant community composition and structure. Increased abundance of *Cocos nucifera*, the coconut palm, drives local (forest stand level) declines in seabirds, and causes a more than 10 fold change in rates of guano addition between palm dominated and dicot dominated forests. Forest composition varies continuously from one forest type to the other, and so does, subsequently, seabird guano input, creating a strong resource gradient across the atoll [35]. While individual stands are highly variable, *C. nucifera* dominated forests tend to have a very different structure, with smaller average stem size (basal area), but higher trunk-level habitat complexity (as measured by stem density and total stand basal area) relative to dicot dominated forests. However, habitat structure variables and subsidy inputs varied continuously and also separately from one another, due to some forests (of both types) being dominated by larger established trees, and others containing smaller and younger trees, as well as a mix of tree types, so we were able to separate out their effects within our statistical analyses. We were thus able to look at the potential of changes in habitat structure as an alternative driver of changes in predator morphology and ecology in this system.

Based on this framework we hypothesized that: 1) geckos in highly subsidized dicot dominated forests would be more abundant, have higher body size and condition (weight per unit length), and feed at a higher trophic position than those in less subsidized forests, 2) the two species would show different strength of response across forest type, with the more coastally associated species, *L.* sp. nov. showing stronger responses to subsidy variation, as its diet is probably more reliant on subsidies (as observed in other lizard species [19], and 3) that *both* changes in subsidy input and changes in forest structure associated with subsidy changes would be important in explaining gecko responses across these forest types.

## Methods

### Study site and species selection

This research was conducted in Palmyra Atoll, in the Northern Line Island chain of the Central Pacific (5°53′N, 162°05′W). The climate is wet tropical (mean temperature 27.5°C; mean annual rainfall ∼4500 mm), with little seasonality. The landmass is approximately 2.5 km^2^ in area, distributed across a ring of small calcium carbonate islets just above sea level. Islet size of surveyed islets varied more than 1000 fold, from 5.29×10^2^ to 6.23×10^5^ m^2^. Plant available soil nutrients varied approximately 100 fold from 8 to 786 µg plant available NO_3_^−^, NH_4_^+^, and PO_4_^−^ g^−1^ dry weight soil. Palmyra is a US National Wildlife Refuge, and the only human settlement is a research station on one islet (not included in this study).

The uninhabited islets of the atoll (16 included in this study) are largely forested, with just five tree species (*C. nucifera*, *Pisonia grandis*, *Pandanus fischerianus*, *Scaevola sericea*, and *Tournefortia argentea*) comprising over 90% of the basal area in these forests [36]. The coconut palm, *C. nucifera*, is the most abundant species, often forming monodominant stands. As *C. nucifera* is typically underused or not used as nesting or roosting habitat by seabirds [35], these stands have greatly reduced marine inputs from guano. Direct estimates of subsidy inputs from seabird guano in *C. nucifera* dominated forests are 23 to 34 kg of N ha^−1^ y^−1^ and 4 to 6 kg of P ha^−1^ y^−1^, as compared to 261 to 653 kg of N ha^−1^ y^−1^ and 42 to 105 kg of P ha^−1^ y^−1^ in dicot dominated forests with low abundance of *C. nucifera* [35]. Marine wrack is likely a relatively small contributor of nutrients to the system and does not vary systematically with forest type [35].

This research focused on two species of geckos of the genus *Lepidodactylus*: *L. lugubris*, and *L.* sp. nov. *L. lugubris* is a widespread asexual species found on islands across the Pacific and Indian Ocean basins, and is thought to have originated from a hybridization event between *L.* sp. nov. and another congeneric species, *L. moestus* [37]. *L. lugubris* and *L.* sp. nov. coexist in the Marshall and Tuamotu Islands, presumably through some form of resource partitioning [33]. From the one system where this was examined, *L.* sp. nov. was more associated with coastal habitats, and it is generally thought to have a narrower diet than the more generalist *L. lugubris* [33]. Since *L.* sp. nov. is more closely tied to coastal habitats, which have greater abundance of birds and thus more subsidies, we expected that the diet of this species would be more strongly influenced by changes in subsidy levels than that of *L. lugubris*, and we expected to observe stronger numerical and behavioral responses to shifts in subsidy inputs in this species, potentially providing it a competitive advantage over its asexual counterpart in highly subsidized environments [10]. As both gecko species are commonly arboreal and are known to have behavioral and competitive responses to changes in habitat structure [24], we also include forest structure variables as potential drivers of observed changes in our analysis.

The two species of *Lepidodactylus* are the only native terrestrial vertebrates on Palmyra, and have no natural predators on the atoll (Supporting Information S1). Both species of *Lepidodactylus* are small (∼45 mm in snout to ventral length (SVL)), but they are easily distinguished in field by variation in foot morphology, and by coloration. They do not differ significantly in levels of interspecific aggression [33]. There are also two introduced vertebrate species, the invasive house gecko, *Hemidactylus frenatus* (largely confined to the one inhabited islet of the atoll, which was not included in this study; Fisher unpublished data), and the invasive rat, *Rattus rattus*, which is abundant on all islets surveyed and shows no change in abundance with change in abundance of palms (Supporting Information S1).

### Vegetation surveys

To determine forest type and assay forest structural characteristics for each of the 16 uninhabited islets studied, vegetation was surveyed across the atoll along a series of 50×2 m coastal transects (n = 51) using methodology described in [36]. On each transect all plants >1 cm DBH were measured and identified; smaller plants were identified but not measured. For analysis of effect of forest type, we grouped islets based on basal area of the coconut palm, *C. nucifera*. A “palm forest” had >75% basal area of *C. nucifera*, while a “dicot forest” had <25% basal area of *C. nucifera*. These breaks coincide naturally with distributional breaks in dominance of these two forest types [36]. Islets with intermediate *C. nucifera* dominance were not used in forest type analyses. Since habitat structure and complexity are known to affect both body condition and foraging behavior of *L. lugubris* [24] as well as many other lizards [27], we calculated three relevant metrics of habitat structure: mean stem size (basal area at breast height), mean stem density (stems per 100 m^2^) and mean basal area (total basal area per 100 m^2^ transect); all values were averaged across all transects on an islet.

### Soil nutrients and islet metrics

Soil nutrient levels (determined for each islet where the geckos were captured) are quantified as the mean plant available NO_3_ and NH_4_ (hereafter referred to as “available soil nitrogen”) and correlate well with measured inputs of bird guano into these systems [5]. This metric was chosen because we assume plant available nitrogen to be the most important limiting nutrient in this atoll system [38], [39]. Soil samples for these analyses were taken on every vegetation transect (minimum of three transects per islet) integrated from 0–20 cm depth. Field moist soil samples were sieved and then immediately extracted in 2 M KCl for NO_3_ and NH_4_ [40]. The extractions were subsequently analyzed using a discrete analyzer (Westco SmartChem 200). The remaining soil after sieving was air dried and used for isotopic analyses of δ^15^N and δ^13^C.

### Gecko abundance, body size, and body condition

Gecko specimens were surveyed and collected in 2008 and 2009 as part of a larger study of biogeography and parasitism in these species (R. Fisher et al. unpublished data). Adult animals of both sexes were collected by hand during the peak activity time for nocturnal geckos (between 2100 and 0000 h) [41], [42]. Animals were collected from a minimum of four sites per forest type, generally within 20 m of the high tide line. To estimate relative abundance at these same sites, we used timed visual encounter surveys (n = 55).

After capture, specimens (n = 169) were identified to species using dorsal skin color patterns and toe shape as described in Hanley et al. 1995 [43]; see also Radtkey et al. 1995 [37]. A subset of the geckos (n = 89) to be utilized in other studies and in stomach content analyses were immediately euthanized. In the lab, specimens were measured (SVL ±0.1 mm) and weighed (±0.01 g). Body condition for each gecko was calculated as mass per unit length [44]. Body condition was not calculated for gravid geckos. Stanford University's Administrative Panel on Laboratory Animal Care approved the use of these animals (Protocol # 23845).

### Stomach content analyses

Gecko stomachs were removed and the contents analyzed under a dissecting microscope by a single observer within 48 hours of being collected in the field. All analyses of diversity and similarity were done by Order, to ensure reliable groupings of partially digested material. The relative percent volume that each type of prey item took up in each stomach was used to determine importance values of prey type. Only stomachs containing at least one identifiable food item were used. Diet diversity and diet similarity were compared both between different gecko species and across forest types (n = 36 in palm forests; n = 53 in dicot forests).

### Prey surveys

Gecko prey items for isotopic analysis were surveyed and collected in both palm and dicot forests via four methods: (1) blacklight traps, (2) sticky traps, (3) pitfall traps, and (4) targeted search efforts (for spiders only). Captured animals were sorted to Order (Hymenoptera was also subdivided into Formicidae and other Hympenoptera; all Isopoda were from family Armadillidiidae) and dried; pitfall trapped insects were then counted and weighed for relative abundance metrics. All prey sampling was conducted in a paired fashion (simultaneous equal sampling in both forest types at equal distances from the coast). For pitfall traps, unbaited traps were set into the ground on 8 islets, with 5 traps in the interior (>10 m from coast; or centermost location of island) and 5 on the coast, for 48 hours each, on three different dates over a 1 month period in 2009. For isotopic analysis, a minimum of 5 animals were sampled, taken at each of 6 islets (3 per forest type; except Formicidae, which were analyzed from only 2 islets per forest type); only legs and (when necessary) head capsules were used for isotopic analysis. Sample size for isotopic analysis by Order is reported in Table S1.

### Stable isotope analyses

Ratios of stable isotopes of both carbon and nitrogen are used in this study as indicators of the trophic position of geckos (δ^15^N), the relative importance of terrestrial vs. marine carbon as an energy source (δ^13^C), and as a tracer of subsidy source (δ^15^N). Consumers generally reflect the stable isotope ratios of their prey with predictable patterns of isotopic fractionation, particularly in the case of nitrogen (roughly 2–3‰ increase in δ^15^N, with each trophic interaction [45]). Carbon isotope ratios strongly reflect the location and pathways by which carbon was fixed, and are clearly different between marine and terrestrial sources (marine sources are enriched in ^13^C compared to terrestrial sources, so that δ^13^C is much more negative in terrestrially fixed carbon sources [23]). The high trophic position (high δ^15^N) and marine feeding habits of seabirds (high δ^13^C) thus allow effective tracking of seabird derived subsidies into the food web [46].

We used gecko tail tips (approximately 1 cm in length) for isotopic analyses, excluding any regenerated tails (n = 164). Tail tips were stored frozen, freeze-dried, ground, lipid extracted, and oven dried prior to analysis [19]. Samples showing post-extraction C∶N values >4.0 (n = 13) were not considered in analyses of δ^13^C levels [47]. Stable isotopic ratios of δ^13^C and δ^15^N were analyzed at the Stanford Stable Isotope Biogeochemistry Laboratory on a Thermo Finnegan Delta-Plus XP IRMS. Using replicate laboratory standards of graphite, ammonium sulfate, and acetelanalide internal, we demonstrated analytical error of less than 0.2‰ for both C and N.

In order to compare trophic position of geckos between forest types that differed in their baseline δ^15^N levels (as a result of differential subsidy contributions by seabirds), we subtracted baseline soil δ^15^N values from gecko δ^15^N measured at the same locations (hereafter called “δ^15^N above soil”; see [35] for details on baseline soil δ^15^N data). Soil δ^15^N levels were analyzed from soil samples taken for soil nutrient analyses. End member isotopic reference values for potential subsidy sources (guano and marine wash), and of plant material, are taken from [35].

### Statistical analyses

We used multiple analyses of variance (MANOVA) to examine effects of species, forest type, and forest type * species interaction on gecko response traits (trophic position, as measured by δ^15^N above soil, δ^13^C, SVL, and body condition). Prior to MANOVA analyses, we tested data sets for normality using a Shapiro-Wilk test, then subsequently checked for homogeneity of variances. When necessary, data were Box-Cox transformed to normalize distributions. Following significant whole model MANOVA, subsequent comparisons across species and treatment were made with Tukey HSD tests. Gecko relative abundance across forest type could not be normalized and was analyzed separately using a non-parametric Wilcoxon ranked-sum test. Significance of all tests was determined to be P<0.05.

In order to understand what factors associated to forest type drive significant changes in gecko response variables (δ^15^N, SVL, and body condition; δ^13^C was not included because it was not found to be significantly correlated with forest type), we constructed three backward stepwise multiple regression models (one for each response variable that was significantly affected by forest type). Factors were removed from model based on Chi Square tests based on AIC_c_ criteria (AIC with second order correction for sample size) and Akaike weights. In each model, we used mean stem size, mean basal area, mean available soil nitrogen, and islet perimeter to area ratio as predictor variables. Mean available soil nitrogen was used as a proxy for subsidy input by birds, a causal relationship documented in [35]. Islet perimeter to area was included to test for variation among islands in the amount marine subsidies (in the form of marine wrack) that could wash ashore and potentially influence terrestrial communities, a factor which has been shown in other studies to influence lizard populations [3]. Islets with high perimeter to area could have a greater proportional input of marine wrack. Prior to all analyses we tested all factors in the model for collinearity using Variance Inflation Factor (R package Car), and found no substantial collinearity.

This regression based approach (as opposed to categorical comparisons used above) allowed us to separate effects of habitat structure and nutrient levels. These explanatory variables both varied by forest type, but varied on different continuous gradients, and were not significantly correlated with each other, which allowed for their orthogonal separation within the regressions and thus prevented habitat structure and nutrient input from being confounded. Except where otherwise stated, all analyses were performed with JMP 8 (SAS Institute, Cary, NC, USA). All graphs/tables depict untransformed data and all mean values are shown with ±1 SD.

To compare gecko diets across forest type we first calculated an importance value for each diet item in each gecko species and each forest type. The importance values were calculated as the sum of the relative abundance (% volume) and relative frequency of each diet item. We compared the diversity and richness of gecko diets (using stomach content data) across sites using Shannon diversity indices (with Hutchenson's modified t-test and significance defined after Bonferroni correction); Order richness curves were generated from a number of richness estimators (Chao, Michaelis-Menten, Jackknife, Bootstrap, ICE) calculated in EstimateS v.8.2 [48], to confirm that we adequately sampled diet diversity in both forest types [49]. Since the richness estimators converged at our observed values, they are not reported. Gecko diet similarity was compared using the Chao-Sorenson similarity index in EstimateS v. 8.2. This index was calculated using abundance data to probabilistically account for rare taxa that may be present in both of the compared samples, but not detected in both of them [50]. We examined diet similarity using nonmetric multidimensional scaling (NMDS, Bray-Curtis similarity coefficient) and analysis of similarities (ANOSIM) on square root transformed data, using relative abundance of all identifiable diet items. We determined significance of the results by Monte Carlo permutations (1000), which are reported as the ANOSIM R statistic calculated in PRIMER [51].

## Results

### Effects of forest type on soil and structural parameters

**Figure 1:**
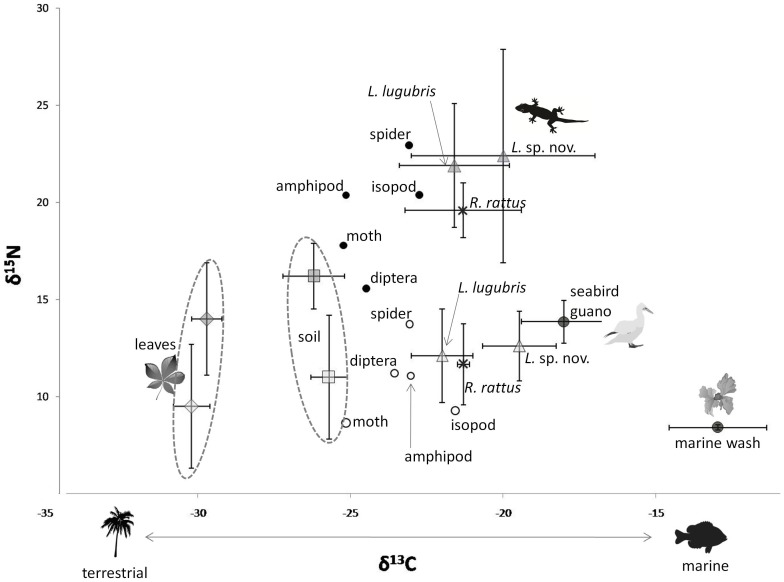
Differences in leaf, soil, and gecko (both *Lepidodactylus lugubris* and *Lepidodactylus* sp. nov.) stable isotope values (means ±1 SD) measured in dicot forests (grey and black symbols) and palm forests (open symbols). Tissues from *R. rattus* in both forest types are shown as a reference (denoted with a *). Prey items (small circles) are shown without SD for visual clarity (SD values are included in Table S1). Significantly enriched δ^15^N values in dicot forests suggest allochthonous subsidies from seabird guano are incorporated in all organisms. Extreme elevation of δ^15^N of both gecko species suggests changes in diet of these species across forest types, or larger changes in food web. Values of δ^13^C in geckos suggest allochthonous marine food sources are important in diets of both gecko species, but are significantly more important for *L.* sp. nov.

Soil nutrients were significantly elevated in dicot forests (NO_3_^−^ = 100.74±26.10 µg/g; NH_4_^+^ = 65.09±5.23 µg/g) as compared to palm forests (NO_3_^−^ = 8.04±2.31 µg/g; NH_4_^+^ = 39.59±6.21 µg/g; n = 57, *P*<0.001 for each). Isotopic values of δ^15^N in soil were significantly different across forest type (Fig. 1; *P*<0.0001) but there were no significant differences in δ^13^C in soils across forest type (see also [35]).

Structural characteristics of forests varied between forest type. Mean stem size was 1.8 times larger in dicot forests (287±146.1 cm^2^ in palm forests, 522.9±121.6 cm^2^ in dicot forests), while stem density was 3.8 times lower (41.9±43.4 stems/100 m^2^ in palm forests, 10.9±4.31 stems/100 m^2^ in dicot forests). Despite smaller mean stem size, the increase in stem density (many, smaller trees) compensated such that there was a 9% higher total basal area in palm forests (0.80±0.15 m^2^ basal area/100 m^2^) than in dicot forests (0.73±0.22 m^2^ basal area/100 m^2^). More details on forest structural difference across forest types are reviewed in [36].

### Effects of forest type on prey communities

**Figure 2:**
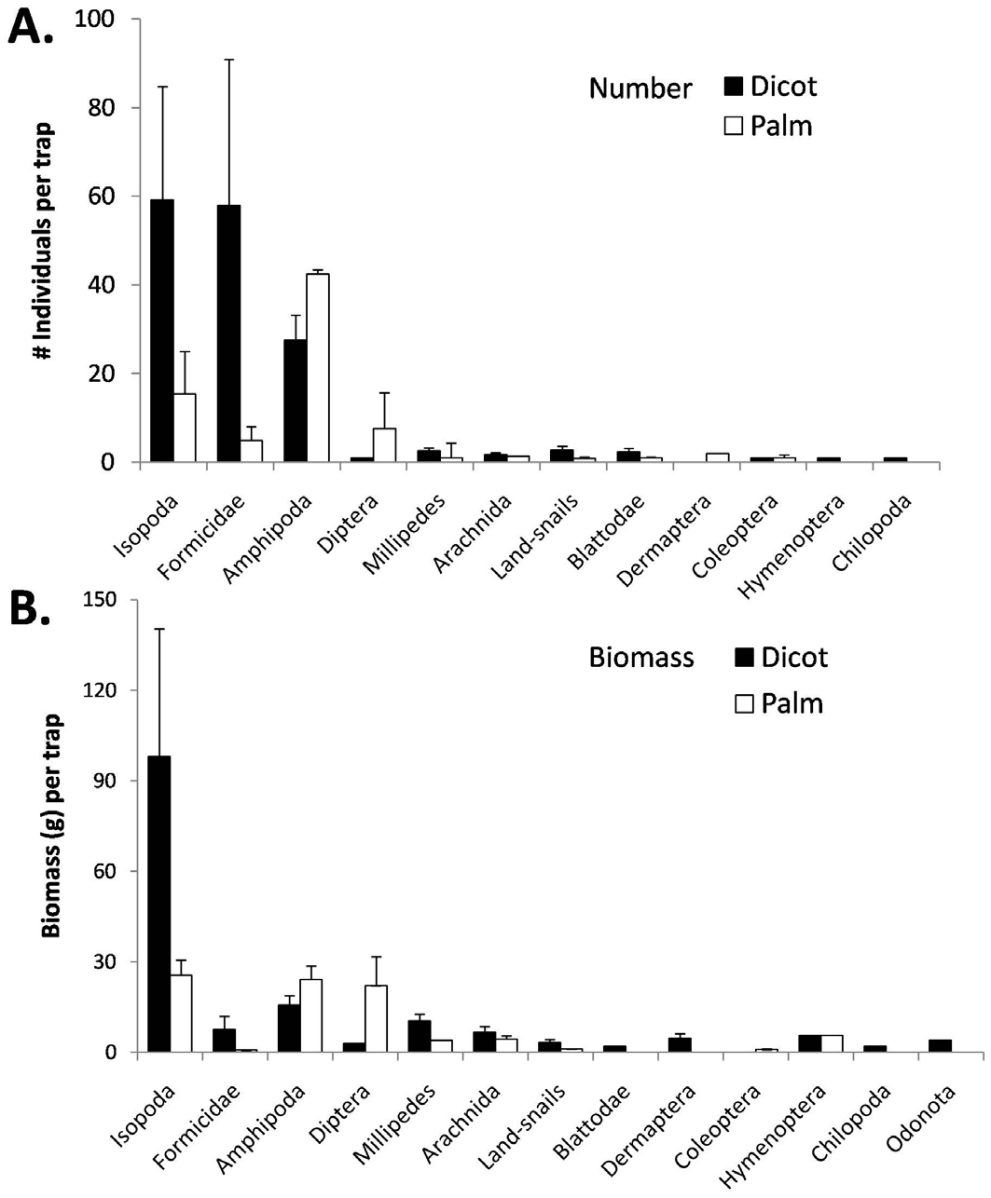
Stomach contents of two species of gecko (*Lepidodactylus lugubris* and *Lepidodactylus* sp. nov.) across forest types. Diet diversity, as estimated by Shannon diversity index, is indicated by forest type. Indices with different letters indicate significant differences based on comparisons with Hutchenson's test; indices that share the same letters are not significantly different from each other.

The three most abundant invertebrate groups in pitfall trapping were Isopoda, Amphipoda, and Formicidae. Formicidae and Isopoda had much higher absolute and relative abundances in dicot forests (39% and 40%, respectively, of all animals caught; Fig. 2) than in palm forests (7% and 20%). Additionally, the large biomass of Isopoda made it by far the greatest component of biomass for both palm (46% of biomass) and dicot (72%) forests. Amphipoda was relatively more important in palm (65% of captures) than dicot forests (18%). There was high variability in both the abundance and composition of invertebrate community across traps and islets. Isotopically, prey items showed extremely high variability among samples, islets, and forest types (Fig. 1; Table S1). While all prey items trended towards higher mean δ^15^N levels in dicot forests than in palm forests, these differences were not always significant and sample size was low for some taxa. Only one prey taxa, Amphipoda, showed significant shifts in δ^13^C levels across forest types, shifting towards less negative δ^13^C values in palm forests (Table S1).

### Effects of forest type and species on gecko response traits

Both forest type (*F*_4,109_ = 48.8, P<0.0001) and species (*F*_4,109_ = 21.1, P<0.0001) affected gecko response traits (δ^13^C, δ^15^N, SVL, and body condition), but there was no significant forest type * species interaction effect (*F*_4,109_ = 0.3, P = 0.87). Given the lack of interaction effect on the whole model, only forest type and species were considered in univariate analyses (Table 1).

**Table 1:** Differences across species and forest type in gecko body size (SVL), body condition, and trophic position, and results of MANOVA.

	*Lepidodactylus lugubris*		*Lepidodactylus* sp. nov.		Forest type		Species	
	Palm	Dicot	Palm	Dicot	F	P	F	P
n	90	30	34	24				
SVL (mm)	37.2±4.9	38.1±2.6	41.1±3.5	40.9±2.1	0.5	0.62	4.4	<0.0001
Body condition (mg/mm)	28±6	34±7	37±6	40±5	5.1	<0.0001	6.6	<0.0001
δ^15^N *	12.1±2.4	21.9±3.2	12.6±1.8	22.4±5.5	–	–	–	
δ^15^N above soil†	0.1±1.8	5.2±1.9	−0.2±1.8	6.0±2.7	14.9	<0.0001	2.6	<0.01
δ^13^C	−22.0±2.2	−21.6±1.8	−19.5±1.2	−20.0±3.0	1.0	0.3	6.4	<0.0001

Values are means ± SD.

* Raw δ^15^N values are presented as reference, but statistics are not calculated for this value as it was not incorporated in whole MANOVA model.

† “δ^15^N above soil” refers to the increase in gecko δ^15^N values over soil δ^15^N values at the same site.

Univariate analyses of response traits show that the two gecko species are distinct in every metric examined (Table 1). *L. lugubris* is smaller (in SVL) than *L.* sp. nov., and has morphometric characteristics or body condition such that it has lower mass per unit body length (Table 1). *L. lugubris* also has significantly more negative δ^13^C values, likely indicating less reliance on marine subsidies. *L. lugubris* also has slightly lower δ^15^N above soil levels, indicating a lower trophic position (Fig. 1). Gecko abundance also varied by species: *L. lugubris* was much more common (0.16±0.45 individuals per minute censusing time) than *L.* sp. nov. (0.05±0.25 individuals per minute).

Besides these species-specific differences, the overarching effects of forest type were the same on both species' body condition and trophic position, with geckos in palm forests having lower SVL, lower body condition, and lower trophic position (as measured by δ^15^N above soil). The shift in δ^15^N across forest types was particularly large, approximately 10‰ for each species; between 5–6‰ of these shifts of δ^15^N could not be explained by changes in soil or plant δ^15^N values (Fig. 1). There was no effect of forest type on gecko δ^13^C. There was also no effect of forest type on gecko abundance for either species (palm = 0.24±0.04 *L. lugubris*, 0.09±0. 04 *L.* sp. nov. individuals per minute; dicot = 0.52±0.26 *L. lugubris*, 0.10±0.09 *L.* sp. nov. individuals per minute).

**Figure 3:**
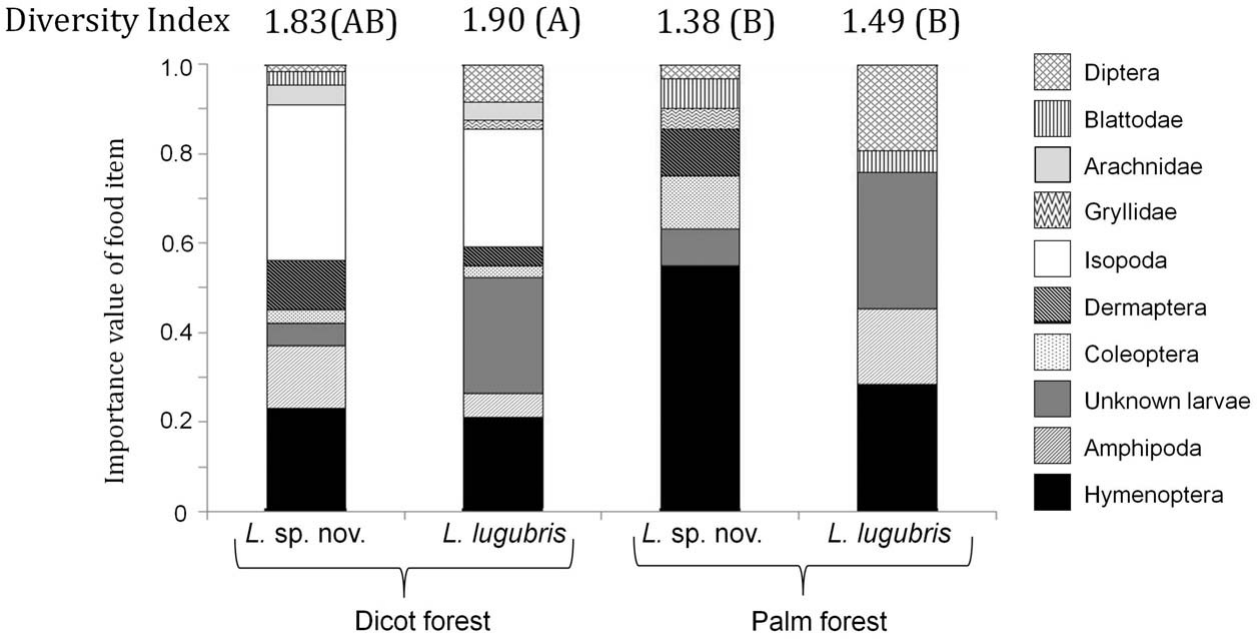
Mean number (A) and dry biomass (B) of invertebrate consumers (+SE) found in pitfall traps placed on coast and interior of 4 islets of each forest type: dicot (black fill) and palm (no fill). Each sample is the average of three collections of an individual trap over 1 month; each collection was from a continuous 48 hour period.

Diet diversity was higher for *L. lugubris* in dicot forests (*t* = 3.0, *P*<0.01). The patterns were similar but only marginally significant for *L.* sp. nov. (*t* = 1.84, *P* = 0.07). Within forest type, there were no significant differences by species (Fig. 3).

Forest type also had an effect on diet similarity. The ANOSIM indicated that diets of *L.* sp. nov. were significantly, but not widely, dissimilar across forest types (*R* = 0.18, *P*<0.001). There was no effect of forest type on the diet of *L. lugubris* (*R* = 0.03, *P* = 0.14). Within a forest type, there was some difference in diets by species in dicot forests (*R* = 0.19, *P* = 0.01), but not in palm forests (*R* = 0.05, *P* = 0.2).

The changes in similarity and diversity are reflected in changes of importance values of specific prey categories for both species, which also shifted between forests (Fig. 3). Isopods were very important to both species of gecko in dicot forests, but were less important or absent from diets of geckos in palm forests. Spiders, though low in overall diet importance, were also consumed more in dicot forests. Hymenoptera were more important in palm forests for both species, as well as Diptera. Differences in prey categories by species and by forest type were also observed (Fig. 3). Some of these results in diet importance values mirrored the results found in the pitfall trap surveys of invertebrate consumers (Fig. 2). For example, both amphipods and flies (Amphipoda and Diptera) were found in higher abundance in the pitfall traps in palm forests, which then showed up in their greater importance value in the diets of geckos in palm forests. The same similarity between elevated pitfall trap abundance and diet importance was found for Isopoda in dicot forests. However, some prey items, like Hymenoptera, bucked this simple correlation. Despite higher abundances of Hymenoptera in dicot forests (Note that in Fig. 2 Hymenoptera is divided into two categories: Formicidae (ants) and other Hymenoptera), they were more important in the diet of geckos in palm forests.

### Drivers of forest type effect

In stepwise multiple regression analysis, the three significant gecko response characteristics analyzed (gecko δ^15^N above soil, SVL, and body condition) had different explanatory predictors (Table 2). Gecko δ^15^N above soil was particularly well explained by the changes in available soil nitrogen, which explained 64% of the total variance. Body condition and SVL were explained less well, but both were primarily explained by mean stem size, with SVL increasing in sites with increased stem size. Islet perimeter to area ratio was not a significant explanatory variable for any response traits.

**Table 2:** Significant predictors of gecko trophic position, body size (SVL), and body condition from a multiple stepwise regression.

Dependent Variable	Significant predictors	SS	F	P	df
1. δ^15^N above soil	Whole model (R^2^ = 0.64)				
	soil nitrogen (+)	1.23	231	<0.0001	1, 127
2. body condition	Whole model (R^2^ = 0.18)				
	mean stem size (+)	0.3	27.2	<0.0001	1, 120
3. SVL	Whole model (R^2^ = 0.13)				
	mean stem size (+)	276	18.5	<0.0001	1, 121

Predictor variables included: available soil nitrogen, mean stem size, mean stand basal area, and islet perimeter to area ratio. After each significant predictor we indicate direction of the correlation as positive (+) or negative (−). As there was no interaction in response between forest type and species, data for both species of *Lepidodactylus* are pooled for this analysis.

## Discussion

Despite very large (>10 fold) differences in subsidy input, and significant changes in habitat structure across forest type [36], we see no evidence of shifts in abundance of these two geckos. This is consistent with the hypothesis that higher level trophic organisms have attenuated responses to subsidies, but is surprising given that other studies of lizards have found strong effects of marine subsidies on abundance [5], [15]. Strong changes in plant community structure along this subsidy gradient may confound the direct bottom-up effects of these subsidies in ways not observed in more arid, and less vegetated sites where other studies were conducted. For example, there may be compensatory changes (increases) in other consumers (i.e. [3]). Also, the periodic nature of subsidy inputs at this site (concentrated during nesting season) may make it more difficult for these lizards to respond numerically to increased subsidy levels. However, we do see important shifts in foraging ecology (diet diversity, diet item importance values, trophic position) and morphological traits (SVL and body condition) across forest types, indicating that a lack of numerical response by predators does not necessarily imply a lack of effect on predator traits, or on food webs.

### Differences between gecko species' response traits

The two gecko species were distinct in both morphological traits and diet. *L. lugubris*, the parthenogenetic species, was smaller and more widespread than *L.* sp. nov., and had an isotopic signature indicative of a more terrestrial (lower δ^13^C), lower trophic level (lower δ^15^N) diet. This is consistent with previous work finding *L.* sp. nov. more confined to coastal habitats, where more marine derived food sources are present [33], and more general observations of strong niche partitioning among sympatric lizard species (i.e [52]). Diet diversity varied among species only in the heavily subsidized dicot forests, further suggesting that the species do partition resources, but perhaps only under certain conditions. The diet similarity between species in palm forests, along with the significant dissimilarity in diet composition for *L.* sp. nov. between forest types could indicate that there are more limited food opportunities in palm forests, while in dicot forests there is room for the two species to specialize. This could be due to greater prey diversity or abundance, frequently observed in subsidized systems [2], [7]; preliminary data supports the suggestion of increased prey diversity and abundance in dicot forests (Young et al., unpublished data).

### Effect of forest type on geckos

As expected, both species respond to changes in forest type, showing larger body size, higher index of body condition, higher trophic position, and greater diet diversity (significantly for *L. lugubris* only) in dicot forests than in palm forests. These responses likely have ecological consequences both for the geckos and for their prey. Increase in body condition, for example, may have strong positive correlation with survival [53], immune response [54], and long term fecundity [55], among other factors. More diverse diets, greater reliance on marine subsidies, and higher trophic position could lead to changes in food web structure, connectivity, and ecosystem function.

The shift in δ^15^N (a proxy for trophic position) between dicot and palm forests was exceptionally large (∼10‰). While some of this change was due to direct incorporation of high δ^15^N guano into the food web, there was still a large and unexplained difference (5–6‰) above the shift seen in soil. The very large shift in δ^15^N we observed strongly suggests that either these predators, or intermediate consumers, or the entire structure of the food web, is fundamentally altered in these systems, despite lack of numerical change in the geckos.

There was, interestingly, no change in δ^13^C across forest type for either species, despite the greater inputs of much less negative δ^13^C guano in dicot forests; this suggests that marine subsidies are likely consumed indirectly by geckos (via incorporation into plants and then plant eating insects) rather than via direct or indirect consumption of guano.

### Interaction of species and forest type

Resource partitioning among sympatric geckos and lizards has driven widespread and much studied genetic and morphological variation and speciation patterns [56]–[57]. Subsidies in particular are hypothesized to drive patterns of richness among lizards [58]. Given the established differences in dependence on spatial subsidies, it seemed likely that the loss of subsidies in palm forests would differentially affect the more subsidized *L.* sp. nov. Contrary to our hypotheses, we found no evidence of interaction between forest type and gecko species. Despite evidence from this study on the greater indirect reliance of *L.* sp. nov. on marine derived sources (based on its less negative δ^13^C and slightly higher δ^15^N values), and the suggestion of others that the stable coexistence between the two species is partially facilitated by a greater dependence of the sexual species on a marine derived diet [33], we observed no difference in the degree of response between species. While reviews have suggested that consumer response may vary based on specific traits of the consumer [10], this conclusion relied on very broad characteristics (e.g. broad feeding habits: insectivore vs. generalist). This study suggests that differential responses may not occur among species with only fine scale differences in reliance on subsidies. However, studies on more diverse species assemblages with more clearly developed differences in subsidy reliance would help further resolve this question.

### Drivers of change in gecko response traits across forest types

Of all responses, change in gecko δ^15^N levels across forest types was the best explained response metric. Increases of δ^15^N in dicot forests appear to be largely explained by the increase in available soil nitrogen in these forests. The extremely large magnitude of the change in δ^15^N across forest types (above soil levels) suggests a 2–3 trophic level shift in gecko diet across forest type. Part of this may be explained by a shift in insect prey base (observed through shifts in relative abundance of prey items in pitfall traps), itself likely driven by higher nutrient levels. However, the relatively small magnitude of diet changes observed suggests that this may be an inadequate explanation for the observed shift. There is no clear alternative food source explaining this difference. While higher amounts of bird guano directly consumed (guanotrophy) at any level in the food chain in dicot forests could potentially explain large changes in δ^15^N (as this change would not be controlled for by changes in soil δ^15^N), the lack of change in δ^13^C across forest types either in geckos or in their prey items suggests that this is not the case; since seabird guano has higher δ^13^C values than terrestrially derived nutrients, changes in proportion of guanotrophy at any point in the foodweb should cause δ^13^C shifts. (Vegetation and microbial communities can both affect soil δ^13^C through a variety of mechanisms [59], which could obscure the marine carbon signal across forest types, and decouple it from the marine nitrogen signal. However, the dissection of that issue is beyond the scope of this paper.)

Furthermore, guano δ^15^N also is not high enough to explain the difference seen between forest types. A possible explanation for the magnitude of the shift may lie in the structure of the food web itself in each forest type. The enriched δ^15^N of both species of gecko above the base soil level in dicot forests indicates that predators may occupy a relatively higher trophic position than geckos in palm forests. This change could be accounted for by changes in the food chain length across forests, or a change in the number of linkages in their respective food webs [20], [60].

The other two metrics that changed significantly across forest type (SVL and body condition) were significantly, but less well explained by our predictor variables, and both were only related to changes in mean stem size. Animals within species were larger and in better body condition in forests with larger average stem size and fewer individual trees, typical of dicot forests. Larger animals may be better able to forage effectively in these open habitats where agonistic interactions are likely to be more common, and may be better able to dominate larger stems [61]–[63]. Given the large body of literature documenting the importance of habitat structure (outside of the context of subsidies) in driving resource partitioning, morphology, and behavior of lizards, it is not surprising that vegetation structure is an important driver in gecko responses. Since change in the plant community is a very common response to subsidy changes, these results suggest that separating effects of habitat changes from that of bottom-up stimuli to food webs may have important implications for understanding how spatial subsidies alter communities. Future work experimentally isolating relative importance of structural and trophic subsidies would greatly help understand the relative importance of these mechanisms.

## Supporting Information

Table S1 Isotopic value of prey and *R. rattus* (mean ± SD) by forest type and order.
(DOCX)

Supporting Information S1 Top predator identity at Palmyra.
(DOCX)
