# Supplementary material for: Effects of Spatial Subsidies and Habitat Structure on the Foraging Ecology and Size of Geckos
Source: PLoS One. 2012 Aug 10;7(8):e41364. doi: 10.1371/journal.pone.0041364 (PMC3416811; doi:10.1371/journal.pone.0041364)
Supplement: Table S1 — Isotopic value of prey and R. rattus (mean ± SD) by forest type and order. (DOCX) [file pone.0041364.s001.docx]

| Consumer | # islets | n | Palm forests | | | | Dicot forests | | | P (t) | | |
| --- | --- | --- | --- | --- | --- | --- | --- | --- | --- | --- | --- | --- |
|  |  |  | | δ^15^N | δ^13^C | δ^15^N | | δ^13^C | δ^15^N | | δ^13^C |  |
| Amphipoda | 3 | 19 | | 10.88 ± 1.08 | -23.00 ± 0.88 | 19.88 ± 2.15 | | -25.36 ± 1.31 | **<0.0001 (10.8)** | | **<0.001 (4.09)** |  |
| Arachnida | 11 | 36 | | 11.58 ± 4.18 | -23.10 ± 0.62 | 23.10 ± 2.79 | | -23.11 ± 1.26 | **<0.001 (6.8)** | | 0.99 (0.0) |  |
| Coleoptera | 3 | 10 | | 7.41 ± 2.27 | -23.94 ± 0.62 | 8.83 ± 5.87 | | -22.97 ± 1.31 | 0.89 (0.1) | | 3.29 (1.09) |  |
| Diptera | 3 | 21 | | 11.34 ± 1.90 | -22.97 ± 1.31 | 14.54 ± 4.87 | | -24.54 ± 0.63 | 0.11 (1.7) | | 13.1 (0.35) |  |
| Formicidae | 2 | 8 | | 14.66 ± 1.81 | -23.82 ± 1.03 | 20.54 ± 3.03 | | -25.19 ± 1.00 | **<0.01 (5.4)** | | 0.03 (3.17) |  |
| Isopoda | 4 | 27 | | 9.43 ± 2.84 | -21.59 ± 0.86 | 20.59 ± 2.02 | | -22.91 ± 2.40 | **<0.0001 (9.2)** | | 13.8 (0.14) |  |
| *R. rattus* | 8 | 49 | | 11.65 ± 3.11 | -21.81 ± 0.32 | 19.37 ± 0.95 | | -21.52 ± 0.78 | **<0.0001 (15.4)** | | 0.29 (0.86) |  |
