# Supplementary material for: Effects of Spatial Subsidies and Habitat Structure on the Foraging Ecology and Size of Geckos
Source: PLoS One. 2012 Aug 10;7(8):e41364. doi: 10.1371/journal.pone.0041364 (PMC3416811; doi:10.1371/journal.pone.0041364)
Supplement: Supporting Information S1 — Top predator identity at Palmyra. (DOCX) [file pone.0041364.s002.docx]

We believe *Lepidodactylus spp.* to be the top predators on the terrestrial ecosystem of Palmyra. There are no passerine birds in the system and seabird and shorebirds are highly unlikely to prey on these arboreal geckos for multiple reasons. First, the primary feeding locations of the birds (in pelagic and intertidal environments) is disparate from locations of geckos (arboreal); second, both species of geckos are nocturnal and the seabird and shorebird species at this site are primarily diurnal; third, direct examination of >500 bird regurgitates yielded no evidence of geckos in their diet (Young unpublished data). The other lizard present at Palmyra, *Hemidactylus frenatus,* is not present on islands used in this study (Fisher unpublished data). The only spider species large enough to potentially predate the geckos at any life stage, *Heteropoda venatoria*, tends to predominate in undergrowth habitat and is more rare in the arboreal spaces where the geckos are common. Its isotopic signature does not indicate that it is at a significantly higher trophic level than the geckos (Supplementary Table 1, Fig 1). Additionally, in more than 100 hours of targeted surveys of spider feeding patterns, there were no observations of spiders feeding on geckos at Palmyra in 2009, 2010, and 2011 (Young observation); it thus seems unlikely that it is a significant threat to the geckos.

The only other potential gecko consumer in the system was *Rattus rattus.* Introduced in the 1950's and likely eradicated from the system in June 2011 (confirmation of eradication will take several years), *R. rattus* has been known to impact lizard populations in other systems [1,2]. However direct examination of rat husking stations (Wegmann unpublished data) and stomach contents (Young et al unpublished data) showed no evidence of gecko predation. Furthermore, for the eight islands for which we collected both gecko and rat tissue, *Lepidodactylus* spp. have a higher mean δ^15^N isotopic value than *R. rattus* (Fig 1; difference between species is not significant) indicating an equal or a potentially higher trophic position to that of *R. rattus* (isotopic sampling detailed below; comparison of *R. rattus* vs. *Lepidodactylus* spp. trophic position used a paired t-test of pooled δ^15^N from all individuals of each taxa sampled on an island).

Even if *R. rattus* was a predator on *Lepidodactylus* spp*,* there is no reason to believe abundance or relative trophic position of *R. rattus* varied across forest types. Trapping efforts (>1400 trap nights, over 11 islands with rats present) documented no significant relationship between abundance of *Cocos nucifera* (as % of canopy cover) and capture rates (rats/trap night) of *R. rattus* abundance (R^2^ = 0.00, P = 0.98; [3] ). These results were repeated in a second trapping effort (Young unpublished data). To look for changes in trophic position, we analyzed isotopic position of muscle tissue from 49 animals sampled on eight islands (≥ 5 animals per island) as part of the previous trapping study (muscle was freeze dried and then powdered). We found no significant relationship between mean *R. rattus* trophic position (above soil δ^15^N) and abundance of *C. nucifera* on a given island (R^2^ = 0.08, P = 0.4). Without these differences in *R. rattus* abundance or trophic position, there should be no differential pressure on geckos across forest types that could potentially explain patterns observed here.

Literature Cited

1. Hoare JM. (2006) Novel predators and naive prey: how introduced mammals shape behaviours and populations of New Zealand lizards. PhD Dissertation, Victoria University Wellington.

2. Hoare JM, Pledger S, Nelson NJ, Daugherty CH (2007) Avoiding aliens: Behavioural plasticity in habitat use enables large, nocturnal geckos to survive Pacific rat invasions. Biol Conserv 136: 510-519.

3. Lafferty KD, Hathaway SA, Wegmann AS, Shipley FS, Backlin AR, Helm J, Fisher RN (2010) Stomach nematodes (*Mastophorus muris*) in rats (*Rattus rattus*) are associated with coconut (*Cocos nucifera*) habitat at Palmyra Atoll. J Parasit 96: 16-20.
